# Attitudes toward innovative mental health treatment approaches in Germany: E-mental health and home treatment

**DOI:** 10.3389/fpsyt.2022.889555

**Published:** 2022-07-15

**Authors:** Lena Lincke, Lisa Ulbrich, Olaf Reis, Elisa Wandinger, Elmar Brähler, Alexander Dück, Michael Kölch

**Affiliations:** ^1^Department for Child and Adolescent Psychiatry, Rostock University Medical Center, Rostock, Germany; ^2^Behavioral Medicine Research Unit, Department of Psychosomatic Medicine and Psychotherapy, Integrated Research and Treatment Center Adiposity Diseases, University of Leipzig, Leipzig, Germany; ^3^Medical Center Department of Psychosomatic Medicine and Psychotherapy, University Medical Center Mainz of the Johannes Gutenberg University Mainz, Mainz, Germany

**Keywords:** eHealth, e-therapy, online interventions, outreach care, representative survey, acceptance of healthcare, attitudes toward healthcare, preferences

## Abstract

E-mental health and home treatment are treatment approaches that have proven to be effective, but are only slowly implemented in the German health care system. This paper explores the attitudes toward these innovative treatment approaches. Data was collected in two large, non-clinical samples representative of the German population in spring 2020 (*N* = 2,503) and winter 2020/2021 (*N* = 2,519). Statistical associations between variables were examined using two-tailed tests. Binary and multinomial logistic regressions were performed to predict attitudes toward online-based treatment concepts and home treatment approaches. Only few (<20%) people preferred online-based treatment approaches, while a larger proportion (~50%) could imagine being treated at home. Overall, younger subjects were more open to online-therapy approaches, while people with lower education preferred more often a traditional therapy setting. Acceptance of online-therapy did not raise significantly during the first months of the COVID-19 pandemic. When different online-based treatment options were available, the probability of accepting home treatment significantly increased with increasing levels of therapeutic support. Further promotion of acceptance for online-therapy and home treatment seems to be necessary. In the future, more information on innovative treatment approaches should be actively provided.

## Introduction

Despite the advanced development of evidence-based treatments for a broad range of mental health problems (1), still a high proportion of people in need of treatment do not receive professional help (2). Within the European Union, about 74% of people with mental disorders stay untreated (3). According to research, various barriers prevent access and use of mental health services: Attitudinal barriers include a little need perceived for treatment (4). The preference to handle the problem on one's own (5) and feelings of shame and embarrassment (6) as beliefs about stigmatization of mentally ill persons have become a global problem for those seeking help (7). In addition, social-structural barriers exist, such as financial obstacles, lack of availability of trained therapists, long waiting lists, unfavorable locations and misinformation about mental disorders (4). With the exception of financial obstacles, barriers to mental health care in Germany are mostly identical compared to other countries. Reasons for the lack of or late onset of treatment in Germany are, for example, lack of knowledge and motivation of the patient, older age, lack of low-threshold psychosomatic and psychotherapeutic offers and regional differences in care with regard to outpatient offers (8).

Two major new developments within mental health care in Germany are (i) home treatment approaches to prevent inpatient treatment and (ii) e-mental health approaches to intensify treatment or to lower barriers for treatment. For the implementation of both treatment approaches in routine care acceptance in the population is a prerequisite.

In home treatment approaches patients are treated in their everyday environment. Home treatment differs conceptually with regard to the acuity of the disease, the frequency and duration of treatment and the composition of the treatment team (e.g., “crisis resolution teams” (9) or “assertive community treatment” (10). Home treatment intends to avoid or shorten inpatient treatment (11, 12). Studies report a high treatment satisfaction among patients and their relatives (11–13). Internationally, home treatment models have become a part of standard psychiatric care in many countries, e.g., in the UK and Norway (14, 15). In Germany home treatment is not yet established in routine care. Although home treatment is strongly recommended in the German guideline for the treatment of severe mental illnesses (16), only a small number of projects provide home treatment (17). Most severely ill patients receive inpatient treatment, as the complex and fragmented organization of the German health care system has long impeded the implementation and financing of home treatment (17, 18). To promote home treatment approaches the social law code has been changed. Since 2018 inpatient equivalent home treatment (IEHT) is a refundable treatment option for mentally ill patients in Germany (§115d SGB V). IEHT refers to an acute psychiatric treatment, which corresponds to inpatient treatment in terms of its complexity and flexibility and is carried out in the patient's home by a multi-professional team, including a psychiatrist (19). Although the number of hospitals offering this form of treatment is slowly increasing, their share is still comparatively low across Germany. More than half of 95 German hospitals surveyed in the year 2020 stated that they definitely did not want to offer IEHT in the future (20). Interestingly the COVID-19 pandemic led to an expansion of home treatment at some hospitals (21). Among the general public, attitudes toward treatment at home seem ambiguous. A recent study found more positive attitudes toward treatment at home among older people, among people who were more comfortable with less social distance from people with mental illness and in regard of one's own treatment compared with the home treatment of others (22).

E-mental health means treatment through the use of the Internet and related technologies such as websites, social media, video conferencing or apps (23, 24). Technological advances and the increasingly ubiquitous Internet access (25) offer new treatment options and have led to increased research interest (26). Latest estimates suggest that more than 10,000 apps for mental or behavioral health are commercially available (27). In recent years, there have been efforts to initiate progress in the field of German digital healthcare through legal changes. The “law for better care through digitization and innovation” (Digitale-Versorgung-Gesetz; DVG), coming into force in 2019, facilitates the implementation of innovative treatment approaches in the digital area by creating new possibilities, e.g., to prescribe digital health applications (DiGAs). According to a report by the National Association of Statutory Health Insurance Funds on the use and development of DiGAs in the period from September 2020 to September 2021, the number of prescriptions was quite low (28). DiGAs are particularly often designed for the area of mental health. In the examined period, half of the existing DiGAs were developed for the treatment of mental disorders (28). The largest frequency of use is also found in this area (29). Currently 14 DiGAs for the treatment of mental disorders are listed in the directory of the Federal Institute for Drugs and Medical Devices, all of them are registered for adults (30). Benefits of e-mental health approaches lie in the opportunity to overcome attitudinal and socio-structural barriers (4, 5). This became even more obvious in times of the COVID-19 pandemic, for which effects of social isolation through quarantine arrangements on mental health have been demonstrated (31, 32). Given the increased number of downloads for mental health apps during the pandemic (33), it has even been described as a “black swan” moment, i.e., as an event permanently shifting mental health care toward online prevention, treatment and care (34). According to a survey conducted by the German Association of Psychotherapists at the beginning of April 2020, 77% of the therapists reported using video treatment, but 95% stated they only started using it since the beginning of the pandemic. This indicates a high willingness to make unexpected, but necessary adjustments to treatment settings in the context of the pandemic (35). Due to its good accessibility, e-mental health approaches have the potential to increase services not only for people living in remote areas, but also for those who are faced with various other barriers to medical care, such as disability or scheduling conflicts. Further advantages are the reduction in attitudinal barriers, such as stigmatization, but also time savings and cost efficiency (2). However, e-mental health services have their own barriers, including concerns about the credibility of online information or the protection of data privacy (36). Furthermore, compared to traditional therapy settings, certain skills, such as computer and internet skills and literacy are required for internet interventions (37). Although anonymity is seen as an advantage when searching for information, the lack of human contact was mainly seen as a disadvantage when coping with and treating a mental problem or mental illness (36). These barriers and disadvantages could have an impact on the acceptance of e-mental health approaches. Several studies reported earlier, that most people would prefer a face-to-face treatment (F2F) compared to e-mental health services (38–40).

E-mental health and home treatment are two treatment approaches that are being slowly implemented in the German health care system. We therefore wanted to examine the attitudes toward these innovative treatment approaches. For this purpose, we evaluated data from a representative survey in the general German population.

## Material and methods

### Recruitment procedure

The analysis is based on two surveys on physical and mental well-being, which were carried out in spring 2020 (S1) and winter 2020/2021 (S2) by an independent market and social research institute (USUMA, Berlin). The aim of the surveys was to collect representative data of the German-speaking resident population in Germany in terms of age, gender, household size, and population by federal state. Data was collected using personally conducted, standardized F2F interviews and a cross-sectional questionnaire. The Ethics Committee of the Faculty of Medicine, University of Leipzig, reviewed and approved both studies (297/16-ek; 474/20-ek).

### Measures

Attitudes toward innovative treatment concepts were assessed with three questions. Attitudes toward online-based treatment concepts were measured within a first question by asking participants which form of therapy they would prefer if they had mental health issues. Answer options were “therapy with a therapist”, “therapy with a therapist, combined with online therapy” (blended therapy) and “pure online therapy”. To examine a possible change in attitudes toward online-based treatment concepts in the course of the COVID-19 pandemic, this question was part of both surveys (S1 and S2). In a second question, participants were asked which form of online therapy they would prefer if they had mental health issues. Answers ranged from “online therapy without therapeutic support”, “online therapy with therapeutic support”, “psychotherapy *via* video conference / Skype” to “I would not use online therapy”. Attitudes toward home treatment approaches were measured by asking participants whether they would like to be treated by a team at home or in their everyday environment if they were mentally ill. Within a 5-point Likert scale, the participants were able to indicate whether the statement is “not applicable at all” (1) to “completely applicable” (5). For the statistical analysis, a binary variable with the values “rejection of home treatment” and “in favor of home treatment” was created.

To examine possible associations between attitudes toward innovative treatment concepts and sociodemographic variables, information on the following parameters was collected: age, gender, educational level, monthly household income and urbanity. The metric variable “age” was divided into meaningful age categories, which are described in Table 1. In order to operationalize urbanity, the residential environment of participants was categorized into rural (≤ 100,000 habitants) and urban areas (>100,000 habitants), according to the definition of the German Federal Institute for Research on Building, Urban Affairs and Spatial Development (32). Furthermore the binary-coded variable “previous psychiatric experience” (“yes”/”no”) and the variable “satisfaction with Internet supply”, measured within a 5-point Likert scale (1 = “not at all satisfied”; 5 = “very satisfied”), served as additional parameters for the analysis of S2.

**Table 1:** Sociodemographic characteristics and response frequencies on other relevant items of the two study samples.

**Variable**		**S1**		**S2**	
		***n***	**%**	***n***	**%**
Sex	Male	1,238	49.8	1,193	47.4
	Female	1,249	50.2	1,322	52.5
	Non-binary^a^			4	0.2
Age	14–25	252	10.1	259	10.3
	26–35	386	15.5	371	14.7
	36–45	388	15.6	397	15.8
	46–55	472	19.0	430	17.1
	56–65	482	19.4	470	18.7
	≥66	506	20.3	592	23.5
	Missings	1	<0.1	0	0.0
School education	Still in school	37	1.5	47	1.9
	Lower education	1,853	74.5	1,899	75.4
	Higher education	595	23.9	551	21.9
	Missings	2	0.1	22	0.9
Income household	Low (<1,250 €)	315	12.7	698	13.9
	Middle (1,250–2,500 €)	1,047	42.1	2,022	40.4
	High (>2,500 €)	1,095	44.0	2,256	45.1
	Missings	30	1.2	30	0.6
Residential environment	<100,000 residents	1,662	66.8	1,637	65.0
	≥100,000 residents	825	33.2	882	35.0
Home treatment^b^	Against	1,243	50.0		
	Pro	1,224	49.2		
	Missings	20	0.8		
Previous psychiatric experience^c^	Yes			295	11.7
	No			2,207	87.6
	Missings			17	0.7
Satisfaction with internet access^c^	Totally unsatisfied			96	3.8
	Unsatisfied			141	5.6
	Neutral			456	18.1
	Satisfied			800	31.8
	Very satisfied			889	35.3
	Missings			137	5.4

a *Categorie was not included in S1*.

b *Item was only part of S1*.

c *Item was only part of S2*.

### Statistical analysis

Data were analyzed using IBM SPSS v.27 (IBM Corp.). In a first step, a frequency analysis was conducted to calculate the percentage of endorsement and rejection of innovative treatment options. Two-tailed tests (Fisher's exact test, Pearson's Chi^2^ test) were used to test for statistical associations between variables of interest. To increase the power of the statistical tests while keeping under control the type 1 error rate, multiple comparisons were adjusted using the Holm-Bonferroni correction (41). Following the recommendations by Agresti (42) for the interpretation of effects of categorical variables, adjusted standardized Pearson residuals (asr) were analyzed to examine the deviations of observed and estimated expected frequencies. Deviations exceeding a value of 2 were considered significant. In a second step, multivariate binary, and multinomial logistic regressions for odds ratio with 95% confidence intervals were conducted to predict attitudes toward innovative treatment concepts. All variables associated with innovative treatment concepts (indicated by an adjusted *p*-value ≤ 0.05) were simultaneously entered as categorical predictors in the equation. Within the first dependent variable, the first (“therapy with a therapist”) and within the second dependant variable, the last category (“I would not use online therapy”) were used as reference categories. “Rejecting toward outreach care” was chosen as the reference category for the third dependent variable.

## Results

Of 5,668 randomly selected target persons who were contacted (5,913 in S2), 44.5% agreed to take part in the survey by giving their written informed consent (43.2% in S2). In total, socio-demographic data were collected of 2,503 subjects in S1 and of 2,519 subjects in S2.

**Figure 1:**
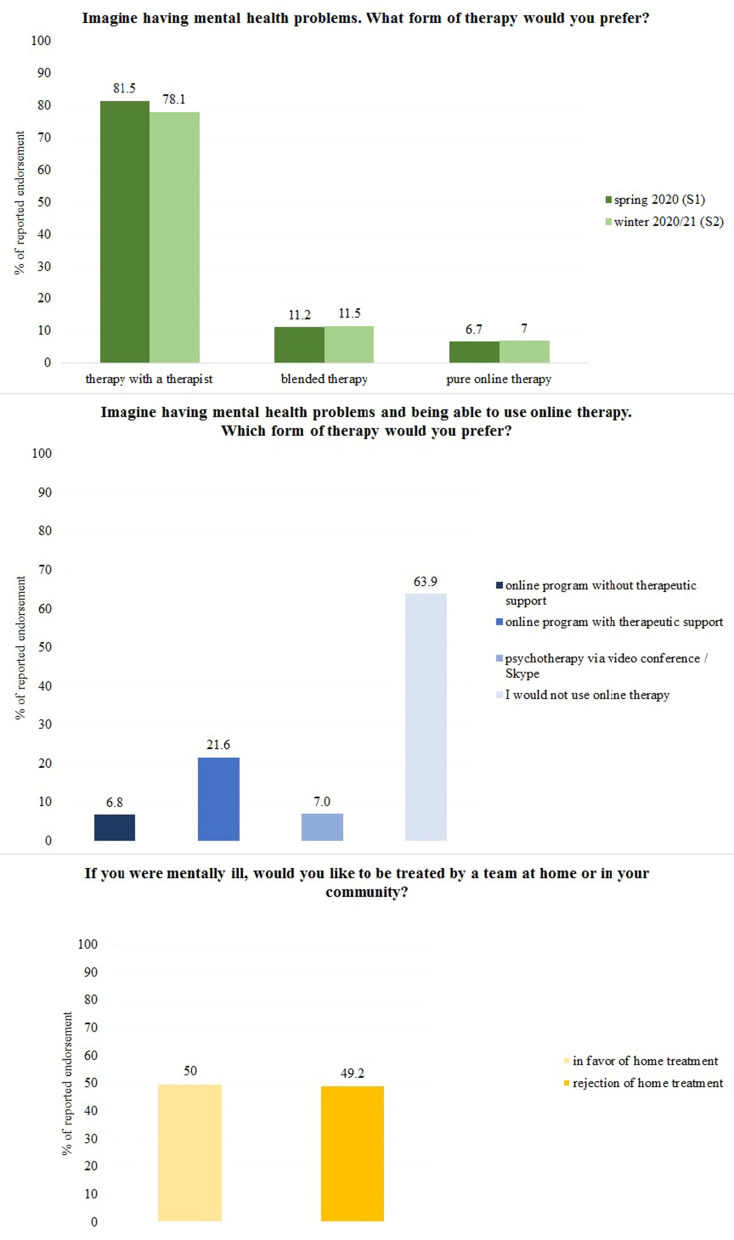
Frequency of responses of participants distributed on the three dependent variables.

After excluding subjects due to inconsistent, illogical answers across the various target items (*n* = 16), the final sample of S1 consisted of 2,487 subjects (50.2% female), aged 14–95 years (*M* = 49.55, *SD* = 17.52). No subjects were excluded for S2, the final sample consisted of 2,519 subjects (52.5% female), aged 16 to 96 years (*M* = 50.33, *SD* = 18.06). Additional sociodemographic characteristics of the samples are reported in Table 1. Figure 1 shows the frequency of responses of participants distributed on the three dependent variables. With regard to attitudes toward online-based treatment concepts, <20% of the participants of S1 preferred an online therapy setting compared to the traditional F2F therapy. Given the choice between different online-based treatment options, more than half of the subjects (63.9%) stated that they would not use online therapy at all. With regard to attitudes toward home treatment approaches, half of the sample stated that they would like to be treated by a team at home or in their community (50%).

For the first dependent variable in S1, Chi^2^ tests and a Fisher-Freeman-Halton-Test showed an association between the preferred form of therapy and sex, age-group, education, income and home treatment. No statistically significant differences were found for urbanity. The detailed data is shown in Table 2. A multinomial logit model predicting attitudes toward preferred form of therapy using sex, age-group, education, income and home treatment as predictors was significant (Nagelkerke's Pseudo *R*^2^ = 0.20). Four out of the five predictors remained significant in the final equation: sex [χ^2^_(2)_ = 24.90, *p* < 0.001], age-group [χ^2^_(10)_ = 222.85, *p* < 0.001], education [χ^2^_(4)_ = 14.13, *p* = 0.007], and home treatment [χ^2^_(2)_ = 47.08, *p* < 0.001]. While being a man, being of younger age, and being a student significantly increased the probability of preferring an online therapy setting rather than a traditional F2F therapy, lower education significantly decreased the probability. Approving home treatment significantly increased the probability of accepting a blended therapy setting, but was also associated with a significant decrease in accepting a pure online therapy rather than a F2F therapy. The detailed model is shown in Table 3. Preferences regarding the form of therapy were also assessed in the second survey (S2). The Chi^2^ tests showed the same results for sex, age-group, education and household income as in the first survey. Furthermore the previous experience with psychiatric treatments was significantly related to the preferred form of therapy [χ^2^_(2)_ = 28.50, adjusted *p* < 0.001, *V* = 0.11]. People who had already experience with psychiatric treatment were underrepresented in terms of their preference for blended therapy (asr: −2.9) or online therapy (asr: −4.2) and preferred significantly more often a therapy with a therapist (asr: 5.1). There was a significant association between the preferred form of therapy and satisfaction with internet access [χ^2^_(8)_ = 21.20, adjusted *p* = 0.021, *V* = 0.07]. People who were very satisfied with their internet access preferred significantly more often blended therapy (asr: 3.0) or online therapy (asr: 2.1). However, there was no statistically significant association between time of survey (S1/S2) and the preferred form of therapy [χ^2^_(2)_ = 1.17, *p* = 0.556, *V* = 0.02].

**Table 2:** Statistical associations between variables of interest of S1.

	**Item I: Type of therapy**					**Item II: Form of online therapy**						**Item III: Home treatment** ^c^			
	**Total sample** **(N** = **2,487)**	**T** **(*****N** =* **2,028)**	**BT** **(*****N** =* **279)**	**OT** **(*****N** =* **166)**	***p*** **-value**	**Total sample** **(*****N** =* **2,487)**	**OP Ø support** **(*****N** =* **168)**	**OP** + **support** **(*****N** =* **536)**	**PT** + **Video** **(*****N** =* **175)**	**Ø OT** **(*****N** =* **1,588)**	***p*** **-value**	**Total sample** **(*****N** =* **2487)**	**Against** **(*****N** =* **1224)**	**Pro** **(*****N** =* **1243)**	***p*** **value**
**Sex**, ***n*** ** (%), sR**															
Male	1,228 (49.7)	972 (79.2), −3.7	143 (11.6), 0.6	113 (9.2), 4.9	<0.001^b^	1,224 (49.6)	112 (9.2), 4.6	255 (20.8), −1.1	81 (6.6), −0.9	776 (63.4), −1.0	<0.001^b^	1,229 (49.9)	646 (52.6), 2.9	583 (47.4), −2.9	0.02^b^
Female	1,245 (50.3)	1,056 (84.8), 3.7	136 (10.9), −0.6	53 (4.3), −4.9		1,243 (50.4)	56 (4.5), −4.6	281 (22.6), 1.1	94 (7.6), 0.9	812 (65.3), 1.0		1,238 (50.2)	578 (46.7), −2.9	660 (53.3), 2.9	
	(missings *n* = 14)					(missings *n* = 20)						(missings *n* = 20)			
**Age groups, *n*** ** (%), sR**															
14–25 y.	250 (10.1)	149 (59.6), −9.7	55 (22.0), 5.6	46 (18.4), 7.8	<0.001^b^	249 (10.1)	39 (15.7), 5.8	78 (31.3), 3.9	27 (10.8), 2.4	105 (42.2), −7.7	<0.001^b^	251 (10.2)	109 (43.4), −2.1	142 (56.6), 2.1	0.357^b^
26–35 y.	386 (15.6)	265 (68.7), −7.4	87 (22.5), 7.6	34 (8.8), 1.8		383 (15.5)	33 (8.6), 1.5	117 (30.5), 4.5	44 (11.5), 3.6	189 (49.3), −6.7		382 (15.5)	175 (45.8), −1.6	207 (54.2), 1.6	
36–45 y.	386 (15.6)	297 (76.9), −2.8	47 (12.2), 0.6	42 (10.9), 3.6		385 (15.6)	37 (9.6), 2.4	119 (30.9), 4.8	39 (10.1), 2.5	190 (49.4), −6.7		383 (15.5)	195 (50.9), 0.5	188 (49.1), −0.5	
46–55 y.	464 (18.8)	394 (84.9), 1.8	41 (8.8), −1.9	29 (6.3), −0.4		467 (18.9)	35 (7.5), 0.6	95 (20.3), −0.8	27 (5.8), −1.2	310 (66.4), 1.0		468 (19.0)	244 (52.1), 1.2	224 (47.9), −1.2	
56–65 y.	481 (19.5)	432 (89.8), 5.0	38 (7.9), −2.6	11 (2.3), −4.3		482 (19.5)	14 (2.9), −3.8	98 (20.3), −0.8	30 (6.2), −0.8	340 (70.5), 3.2		481 (19.5)	240 (49.9), 0.1	241 (50.1), −0.1	
>65 y.	505 (20.4)	490 (97.0), 9.9	11 (2.2), −7.3	4 (0.8), −6.0		500 (20.3)	10 (2.0), −4.8	29 (5.8), −9.7	8 (1.6), −5.4	453 (90.6), 13.7		501 (20.3)	261 (52.1), 1.2	240 (47.9), −1.2	
	(missings *n* = 15)					(missings *n* = 21)						(missings *n* = 21)			
**School education, *n*** ** (%), sR**															
students	35 (1.4)	16 (45.7), −5.6	8 (22.9), 2.2	11 (31.4), 5.9	<0.001^a^	36 (1.5)	9 (25.0), 4.4	14 (38.9), 2.5	4 (11.1), 1.0	9 (25.0), −5.0	<0.001^b^	37 (1.5)	14 (37.8), −1.4	23 (62.2), 1.4	0.204^b^
Lower	1,844 (74.6)	1,566 (84.9), 6.5	164 (8.9), −6.5	114 (6.2), −1.8		1,839 (74.6)	114 (6.2), −2.1	357 (19.4), −4.8	100 (5.4), −5.4	1,268 (69.0), 8.1		1,836 (74.5)	935 (50.9), 2.2	901 (49.1), −2.2	
Higher	592 (24.0)	444 (75.0), −5.1	107 (18.1), 6.0	41 (6.9), 0.2		590 (23.9)	45 (7.6), 0.9	165 (28.0), 4.2	70 (11.9), 5.2	310 (52.5), −6.9		592 (24.0)	274 (46.3), −1.9	318 (53.7), 1.9	
	(missings *n* = 16)					(missings *n* = 16)						(missings *n* = 22)			
**Household income, *n*** ** (%), sR**															
<1,250 €	312 (12.8)	262 (84.0), 1.0	33 (35.2), −0.4	17 (5.4), −1.0	0.018^b^	311 (12.8)	21 (6.8), −0.1	60 (19.3), −1.1	16 (5.1), −1.5	214 (68.8), 1.8	<0.001^b^	312 (12.8)	151 (48.4), −0.4	161 (51.6), 0.4	0.836^b^
1,250–2,500 €	1044 (42.7)	882 (84.5), 2.8	95 (9.1), −3.0	67 (6.4), −0.6		1,036 (42.5)	60 (5.8), −1.8	185 (17.9), −4.0	63 (6.1), −1.7	728 (70.3), 5.3		1,042 (42.7)	513 (49.2), −0.3	529 (50.8), 0.3	
>2,500 €	1,088 (44.5)	859 (79.0), −3.5	148 (13.6), 3.2	81 (7.4), 1.2		1,091 (44.7)	86 (7.9), 1.8	285 (26.1), 4.7	95 (8.7), 2.7	625 (57.3), −6.5		1,085 (44.5)	544 (50.1), 0.5	541 (49.9), −0.5	
	(missings *n* = 43)					(missings *n* = 49)						(missings *n* = 48)			
**Urbanity, *n*** **(%), sR**															
<100.000	1,653 (66.8)	1,366 (82.6), 1.2	173 (10.5), −1.8	114 (6.9), 0.5	0.179^b^	1,649 (66.8)	116 (7.0), 0.6	345 (20.9), −1.4	113 (6.9), −0.7	1,075 (65.2), 1.2	0.427^b^	1,646 (66.7),	803 (48.8), −1.2	843 (51.2), 1.2	0.486^b^
≥100.000	820 (33.2)	662 (80.7), −1.2	106 (12.9), 1.8	52 (6.3), −0.5		818 (33.2)	52 (6.4), −0.6	191 (23.3), 1.4	62 (7.6), 0.7	513 (62.7), −1.2		821 (33.3),	421 (51.3), 1.2	400 (48.7), −1.2	
	(missings *n* = 14)					(missings *n* = 20)						(missings *n* = 20)			
**Home treatment, *n*** ** (%), sR**															
Against	1,218 (49.6)	1,037 (85.1), 4.1	82 (6.7), −7.1	99 (8.1), 2.7	<0.001^b^	1,216 (49.6)	90 (7.4), 1.1	176 (14.5), −8.7	56 (4.6), −4.7	894 (73.5), 9.5	<0.001^b^				
Pro	1,240 (50.4)	977 (78.8), −4.1	196 (15.8), 7.1	67 (5.4), −2.7		1,235 (50.4)	77 (6.2), −1.1	359 (29.1), 8.7	117 (9.5), 4.7	682 (55.2), −9.5					
	(missings ***n*** = 29)					(missings ***n*** = 36)									

*sR, standardized Pearson residual; T, therapy with therapeut; BT, blended therapy; OT, online therapy; OP Ø support, online-program without therapeutic support; OP + support; online-program with therapeutic support; PT + video, psychotherapy via video/skype; Ø OT, no online therapy*.

a *Fisher's exact test;*

b *Pearson's χ^2^test*.

c *associations between home treatment and item I and item II were calculated, but have been omitted because of redundancy*.

**Table 3:** Factors associated with attitudes toward online-therapy: multinomial logistic regressions.

		**Item I: Type of therapy**				**Item II: Form of online therapy**					
		**Blended therapy** **(therapy with therapist**^a^**)**		**Online therapy** **(therapy with therapist**^a^**)**		**Online-progr. without therapeutic support** **(no online therapy**^a^**)**		**Online-progr. with therapeutic support** **(no online therapy**^a^**)**		**Psychotherapy** ***via*** **video/skype** **(no online therapy**^a^**)**	
		**OR**	**95% CI**	**OR**	**95% CI**	**OR**	**95% CI**	**OR**	**95% CI**	**OR**	**95% CI**
**Sex (female^*a*^)**											
male		1.14	0.87, 1.48	2.38^***^	1.67, 3.38	2.07^***^	1.46, 2.92	0.95	0.77, 1.17	0.89	0.64, 1.23
**Agegroup (>65 y.^*a*^)**											
14–25 y.		12.70^***^	6.31, 25.56	37.64^***^	13.00, 108.95	13.48^***^	6.29, 28.90	9.49^***^	5.68, 15.87	11.46^***^	4.90, 26.83
26–35 y.		12.31^***^	6.40, 23.69	16.50^***^	5.76, 47.27	7.47^***^	3.58, 15.57	9.12^***^	5.76, 14.42	10.96^***^	5.00, 24.05
36–45 y.		6.06^***^	3.06, 12.04	18.70^***^	6.57, 53.22	8.48^***^	4.08, 17.62	9.50^***^	5.99, 15.07	9.61^***^	4.32, 21.38
46–55 y.		4.07^***^	2.04, 8.11	9.57^***^	3.30, 27.76	4.86^***^	2.34, 10.09	4.56^***^	2.88, 7.21	4.35^***^	1.92, 9.86
56–65 y.		3.58^***^	1.80, 7.14	3.15	0.99, 10.02	1.76	0.77, 4.03	4.44^***^	2.83, 6.97	4.71^***^	2.12, 10.48
**School education (higher education^*a*^)**											
Students		0.98	0.36, 2.64	2.69^*^	1.04, 6.98	3.02	0.97, 9.42	2.25	0.83, 6.08	0.99	0.23, 4.24
Lower education		0.63^**^	0.47, 0.84	1.09	0.73, 1.62	0.88	0.60, 1.30	0.75^*^	0.59, 0.96	0.49^***^	0.34, 0.70
**Household income (>2,500 €^*a*^)**											
<1,250 €		0.82	0.53, 1.27	0.81	0.45, 1.46	0.88	0.51–1.51	0.81	0.57, 1.15	0.65	0.36, 1.17
1,250–2,500 €		0.83	1.77, 3.10	1.13	0.79, 1.63	0.85	0.58–1.23	0.74^*^	0.58, 0.94	0.81	0.57, 1.17
**Home treatment acceptance (against^*a*^)**											
Pro		2.34^***^	1.77, 3.10	0.68^*^	0.48, 0.95	1.18	0.84–1.64	2.79^***^	2.24, 3.47	2.82^***^	2.00, 3.98
Model fitting: *χ*^2^ (df)					362.31 (22)^***^						503.90 (33)^***^
Goodness of fit	(Pearson) χ^2^ (df)				282.22 (272)						400.02 (408)
	(Deviance) χ^2^ (df)				269.77 (272)						411.11 (408)
Pseudo-*R^2^* (Nagelkerke's)					0.20						0.22

*OR, odds ratio; 95% CI, 95% confidence interval; df, degree of freedom;*

* *p < 0.05;*

** *p < 0.01;*

*** *p < 0.001;*

a *reference*.

For the second dependent variable, Chi^2^ tests showed an association between the preferred type of online therapy and sex, age-group, income, education and home treatment. No statistically significant differences were found for urbanity. The detailed data is shown in Table 2. A multinomial logit model predicting attitudes toward preferred type of online therapy using sex, age-group, education, income, and home treatment as predictors was significant (Nagelkerke's Pseudo *R*^2^ = 0.22). Four out of five predictors remained significant in the final equation: sex [χ^2^_(3)_ = 20.65, *p* < 0.001], age-group [χ^2^_(15)_ = 262.34, *p* < 0.001], education [χ^2^_(6)_ = 23.18, *p* = 0.001], and home treatment [χ^2^_(3)_ = 109.26, *p* < 0.001]. With regard to household income, there were inhomogeneous effects. The relationship between income and form of online therapy in the overall model was not significant [χ^2^_(6)_ = 8.15, *p* = 0.23]. However, one level of the predictor “household income” showed a significant effect, as described below: While being a man and of younger age significantly increased the probability of preferring some type of online therapy rather than no online therapy, lower education and an income between 1,250 and 2,500€ significantly decreased the probability. Interestingly, approving home treatment only significantly increased the probability of accepting an online program with therapeutic support or a psychotherapy *via* video conference/skype rather than no online therapy. The detailed model is shown in Table 3.

For the third dependent variable, Chi^2^ tests showed an association between the acceptance of home treatment and sex, preferred form of therapy and preferred type of online therapy. No statistically significant differences were found for age-group, education, income and urbanity. The detailed data is shown in Table 2. A binary logit model predicting attitudes toward the acceptance of home treatment using sex, preferred form of therapy, and preferred type of online therapy as predictors was significant. While being a woman significantly increased the probability of accepting home treatment rather than rejecting home treatment, preferring a pure online therapy compared to a traditional F2F therapy significantly decreased the probability. Given the choice of different kinds of online-based treatment options, the probability of accepting home treatment significantly increased with increasing levels of therapeutic support. The detailed model is shown in Table 4.

**Table 4:** Factors associated with attitudes toward home treatment: binary logistic regression.

	**Item III: home treatment**	
	**pro (against** ^a^ **)**	
	**OR**	**95% CI**
**Sex (female** ^*****a*****^ **)**		
male	0.81^*^	0.69–0.96
**Type of therapy (therapy with therapist** ^***a***^ **)**		
Blended therapy	1.13	0.81–1.58
Online therapy	0.41^***^	0.27–0.61
**Form of online therapy (no online therapy** ^***a***^ **)**		
Online-progr. without therapeutic support	1.90^**^	1.27–2.84
Online-progr. with therapeutic support	2.78^***^	2.15–3.59
Psychotherapy *via* video/skype	3.02^***^	2.11–4.30
Model fitting: χ^2^ (df)	145.12 (6)^***^	
Hosmer-Lemeshow-Test	2.26 (6)	
Pseudo-*R^2^* (Nagelkerke's)	0.08	

*OR, odds ratio; 95% CI, 95% confidence interval; df, degree of freedom;*

* *p < 0.05;*

** *p < 0.01;*

*** *p < 0.001;*

a * reference*.

## Discussion

People in Germany show rather low acceptance rates toward the innovative mental health treatment forms examined, especially toward e-mental health. Conventional F2F therapy seems to stay the preferred form for most people. Interventions with some degree of therapeutic support are preferred to pure online therapy. Acceptance of e-therapy did not raise significantly during the first months of the COVID-19 pandemic, although many people in Germany have broadened their experience with online tools, as they have been forced to work from home (43). However, the date of our second survey was only half a year after the start of the pandemic, so that long-term effects could not yet be mapped.

Due to the small effect size (Nagelkerke's Pseudo *R*^2^ = 0.08) the relationship between the attitude toward home treatment and the recorded sociodemographic characteristics should be interpreted with caution. Regardless of the sociodemographic differences, the only partial acceptance of home treatment in the general population contrasts with the high acceptance among patients who already have experience with home treatment: they tend to evaluate it positively (11, 13, 44) and even show increased treatment satisfaction compared to patients who received standard treatment (11, 12). Consequently, the positive attitude seems to be highly dependent on personal experience (45). Aside from these findings on patient attitudes, attitudes in the general population appear to be mixed and dependent on factors such as the type of diagnosis. In a study from the UK that used case vignettes to ask people about their attitudes toward home treatment, the participants expressed their support for this form of treatment for patients with depression or alcohol abuse. At the same time, participants were of the opinion that people with a drug addiction or schizophrenia should not be treated at home. Whether the participants themselves had a mental illness turned out to be irrelevant for their assessment (22). Our data also showed different attitudes toward home treatment. Home treatment approaches, when compared to inpatient treatment, have been shown to be just as effective in reducing symptoms (16) and help reduce hospitalization and treatment discontinuations (11, 12). Half of the participants stated that they would not consider to be treated at home if they had a mental illness. Other aspects of home treatment may dominate these people's perceptions: they may be reluctant to open the own home to health care providers or fear stigmatization if neighbors notice that treatment teams visit them at home. In the German health care system home treatment for psychiatric patients is rather an exception: currently the majority of patients are treated in inpatient settings or by residents (20). The gradual implementation of home treatment in Germany may lead to a higher acceptance in the future, since attitudes toward home treatment are strongly dependent on experience.

Issues of privacy, minimization of stigma due to treatment and improvement of access to care are aspects which are discussed as positive factors of e-mental health (2). Given the fact that people asked for their preference mainly prefer the well-known conventional care forms of therapy may indicate that these factors are not the most substantial factors for people in their decision which treatment they would prefer. In fact, while participants in a previous study rated anonymity as a very important aspect, they still perceived it as the least important of 12 different areas of mental health treatment expectations (46). Interestingly, however, there seem to be people for whom the aspect of anonymity and distance in the treatment setting seems to be attractive. In this regard, our analysis showed that people who prefer pure online therapy to F2F therapy are also less open to home treatment. The low rate of people who prefer e-therapy is in line with former studies, where the preference for e-mental health services only ranged from 1.2% (47) to 29.6% (48), while between 32% (49) and 96.4% (50) preferred F2F treatment. Furthermore, our data showed that people who have prior experience with psychiatric treatment are more skeptical about online-therapy approaches than those who have no previous experience. This is consistent with findings from a UK survey in which respondents who stated that they had sought help for mental health problems in the past reported significantly lower intentions to use e-therapy in the form of smartphone apps in the future (46). Patients may have experienced the relationship with the therapist as an important factor in their treatment and may fear that it may not be as easily established in an online setting. In a qualitative survey of a large German health insurance company on user satisfaction with DiGAs, some of the users complained about the lack of personal contact with their doctors and therapists (29). Indeed, the therapeutic relationship is seen as an important non-specific factor in psychotherapy research (51) and contributes significantly to the therapeutic success. It has been defined as “an emotional bond between the client and therapist, characterized by warmth, trust and empathy, and agreement on the goals and tasks of the intervention” [Bordin, 1994 as cited in (52)]. According to current meta-analytical evidence, therapeutic alliance and outcome are significantly correlated not only in F2F-therapy (53–55), but also in an e-therapy setting (56), indicating that fostering a stable alliance is important to promote efficacy of treatment across all therapy settings (53, 56). However, it is questionable whether it is more difficult to establish a therapeutic alliance in an e-therapy setting than in F2F-contact. This does not seem to be the case, at least from a client perspective: Across various studies, it has been shown that patients evaluate the therapeutic relationship in e-therapy interventions on average about as well as in F2F-therapies (57–59). However, it should be noted that this refers to e-therapy interventions which include some level of support from a therapist and, in general, ratings of the therapeutic alliance are lower in unsupported interventions (60). The incorporation of specific design features that create the impression of a bidirectional therapeutic relationship, as well as interventions tailored specifically to different patients using digital phenotyping and machine learning approaches, offer much potential to find future ways to cultivate a strong therapeutic alliance that is independent of human support (60). However, a therapeutic relationship currently seems easier to establish when some level of human support is involved. Blended interventions may represent a promising compromise as they combine F2F-contacts with a therapist with e-therapy sessions. Therapists prefer blended therapy to therapies conducted entirely *via* the Internet (61). Our findings indicate, that a blended approach may also find greater acceptance among the general population: in our survey openness to e-therapy increased with an increasing degree of support from a therapist. Similar to our results in 2020–2021, a preference for therapist-assisted e-mental health interventions over unguided web-based programs was found by Casey et al. in 2013 (62). Overall, the relationship with a therapist through direct contact seems to be very important for people when considering treatment of mental illness (63). This is also consistent with our finding that the probability of preferring home treatment increased significantly with increasing therapeutic support of the preferred online-based treatment option. According to this, certain people are characterized by an openness to various innovative treatment approaches, as long as personal contact with a therapist is maintained.

In terms of socio-demographics, specific groups may be easier to target with e-therapy approaches than others. We found that younger people and people with higher education are more likely to accept e-therapy. Already Eichenberg et al. found in 2013 that especially younger persons, people with an university degree and people with higher incomes show a higher readiness to use media-assisted psychotherapy (38). This population would therefore be a target group for e-mental health care, which is relevant for current projects dealing with the implementation of e-therapy interventions (e.g., DRKS00022420). The fact that younger people show higher acceptance for e-therapy is an opportunity for child and adolescent psychiatric and psychotherapeutic services: especially low threshold offers for service could reach a high number of youths and young adults. Transition is a crucial issue in mental health service (64). Once they reach an upper age limit (typically at age 18, 18–21 years in Germany), young patients can no longer be treated by child and adolescent mental health services and need to be transferred into adult services. This transition often leads to difficulties with regard to the continuity of mental health care (65–67). Patients who had to leave child and adolescent care due to their age describe barriers to transition such as a lack of knowledge about where else they could access care, difficulties with finding a service suited to their level of need and long waiting times to access care from a new provider (68). Due to these and other barriers, some patients fail to transition to adult mental health services at all (66). In the absence of other alternative, these patients are often referred to general practitioners (65). However, these often do not have sufficient expertise to treat mental illnesses, which is also perceived as such by patients and can discourage them from discussing their mental health needs (65). E-therapy could support transition, especially since we found a relatively high acceptance for e-mental health approaches in this age group. This could be an interesting starting point for a tailored treatment approach for these often hard to reach group (69). E-therapy approaches specially tailored to transition age youth could avoid the problem that adult mental health services are often not optimally matched to the developmental stage of young people (70). Additionally, e-therapy applications, such as mental health apps, may be prescribed by medical practitioners which lack the expertise to treat patients with mental disorders themselves (e.g., general practitioners), but nevertheless often have responsibility for the care of these young patients. In this way, specialized offers could be made accessible to transitioning patients who are not yet supported by adult mental health services.

## Limitations and future directions

Our study had several limitations. The wording of the questions in the questionnaire was quite general: The concept of home treatment was explained very briefly, but no further explanation was given on the different forms of e-therapy (*via* video conference, with or without therapeutic support). It is possible that some of the respondents indicated a preference for a classic treatment setting because they could not envision the other options (71). As a more detailed questionnaire with more explanations could have resulted in more differentiated answers, therapy settings should be described in more detail in future surveys. Furthermore, as noted by other authors, questionnaire surveys should not be used as the only option to assess public acceptability of innovative mental health treatments (71). Qualitative methods have the advantage that the reasons for accepting or rejecting certain treatment settings can be recorded in a differentiated manner. In our current survey, we found that a significant part of the German population is skeptical about innovative mental health therapy offers. However, based on the data, no conclusions can be drawn as to what caused this rejection. It is important to note that possible explanations—such as the expectation of a poorer therapeutic relationship in an e-therapy setting—are speculative. Qualitative methods could help to better understand why innovative therapies are rejected and to find ways to make them more attractive to a broader group of people. Since innovative therapy offers with therapeutic support seem to be more widely accepted, a more detailed examination of the preferences of e-health applications explicitly as add on would also be interesting. Further development of e-mental health offers should take into account that blended therapies not only show higher adherence (72), but are also preferred by most people to pure online therapy without contact with a therapist.

Although we asked in the second survey whether the participants had previous experience with psychiatric treatment, it was not recorded what exactly this previous experience looked like. Other studies have shown that the attitude toward innovative mental health offers is dependent on previous experience (45). Future surveys should specifically record personal experience with e-therapy or home treatment in order to check whether this influenced their attitude toward these approaches.

Promotion of acceptance is particularly necessary for people who are rather reluctant to use e-therapy and home treatment. Research shows that a large part of the Germans is unaware of the diverse possibilities offered by the Internet today (71). However, knowledge about the procedure or the setting of online therapy increases its acceptance (62, 73). Therefore, information on e-mental health and home-treatment may help to increase acceptance, which would be beneficial if these forms of therapy are to be implemented more extensively in the German healthcare system. According to our results, special groups should be specifically addressed with information (people with a low level of education and older people). This would be necessary if e-mental health care interventions shall help to improve care in rural regions. Otherwise, the benefits of e-therapy and the contribution to an improved mental health care will remain small. Since we found that the preference for online therapy increased with a high level of satisfaction with one's own internet supply, network coverage is also relevant for the implementation of e-mental health.

There were hardly any clear associations between home treatment and the sociodemographic characteristics examined. However, the relationship should be re-examined in future studies. Since novel approaches to home treatment of eating disorders are currently being investigated and implemented in Germany (74, 75), it is of great interest to understand by whom they might be accepted.

With regard to the influence of the COVID-19 pandemic, it would be interesting to investigate long-term effects. Even if the first few months of the COVID-19 pandemic did not lead to a change in attitude toward e-therapy, this may have changed in the meantime.

## Conclusion

The aim of our study was to examine the current attitudes of the German population toward home treatment and e-therapy. While almost half of the participants expressed openness toward home treatment approaches, the majority were skeptical about e-therapy. Attitudes toward e-therapy did not change during the first months of the COVID-19 pandemic. The vast majority indicated that they prefer a classic F2F-treatment setting. E-therapy offers with the support of a therapist were preferred to unsupported e-therapy. E-health may therefore be rather accepted as an additional tool within psychotherapy to intensify therapy than a single method without contact to a therapist. Results underline the high need for informational campaigns about treatment options, especially for groups with high skepticism, like older people and people with lower education. Despite the relatively high level of skepticism in the general population, certain groups might particularly benefit from the implementation of e-therapy. Since younger people are more receptive to the approach, e-therapy could help to optimize transition processes in mental health care. Persons with previous mental health care experience reject e-therapy more strongly than persons without this experience. E-mental health could therefore be particularly suitable for people who are coming into contact with the psychiatric care system for the first time and offering a low-threshold entry point into psychiatric treatment. Health care in Germany is mainly planned and designed by service providers, health insurances and politicians, but rarely includes the treatment preferences of patients or the general population in the development of the system. In order to change this in the future, further studies are necessary, which examine the preferences of patients and the general population in a differentiated manner and explain which treatment would be accepted by whom.

## Data availability statement

The raw data supporting the conclusions of this article will be made available by the authors, without undue reservation.

## Ethics statement

The studies involving human participants were reviewed and approved by Ethics Committee of the Faculty of Medicine, University of Leipzig. Written informed consent to participate in this study was provided by the participants' legal guardian/next of kin.

## Author contributions

EB, OR, LL, and MK contributed to conception and design of the study. LL and LU performed the statistical analysis and wrote the first draft of the manuscript in consultation with OR, EW, EB, AD and MK. MK finalized the manuscript. All authors contributed to manuscript revision, read, and approved the submitted version.

## Conflict of interest

The authors declare that the research was conducted in the absence of any commercial or financial relationships that could be construed as a potential conflict of interest.

## Publisher's note

All claims expressed in this article are solely those of the authors and do not necessarily represent those of their affiliated organizations, or those of the publisher, the editors and the reviewers. Any product that may be evaluated in this article, or claim that may be made by its manufacturer, is not guaranteed or endorsed by the publisher.
